# Tabetri™ (*Tabebuia avellanedae* Ethanol Extract) Ameliorates Atopic Dermatitis Symptoms in Mice

**DOI:** 10.1155/2018/9079527

**Published:** 2018-03-15

**Authors:** Jae Gwang Park, Young-Su Yi, Sang Yun Han, Yo Han Hong, Sulgi Yoo, Eunji Kim, Seong-Gu Jeong, Adithan Aravinthan, Kwang Soo Baik, Su Young Choi, Jung-Il Kim, Young-Jin Son, Jong-Hoon Kim, Jae Youl Cho

**Affiliations:** ^1^Department of Genetic Engineering, Sungkyunkwan University, Suwon 16419, Republic of Korea; ^2^Department of Pharmaceutical Engineering, Cheongju University, Cheongju 28503, Republic of Korea; ^3^College of Veterinary Medicine, Chonbuk National University, Iksan 54596, Republic of Korea; ^4^R&I Planning Department, Nutribiotech Co. Ltd., Seoul 06132, Republic of Korea; ^5^Department of Information Statistics, Kangwon National University, Chuncheon 24341, Republic of Korea; ^6^Department of Pharmacy, Sunchon National University, Suncheon 57922, Republic of Korea

## Abstract

*Tabebuia avellanedae* has been traditionally used as an herbal remedy to alleviate various diseases. However, the plant's pharmacological activity in allergic and inflammatory diseases and its underlying mechanism are not fully understood. Therefore, we investigated the pharmacological activity of Tabetri (*T. avellanedae* ethanol extract (Ta-EE)) in the pathogenesis of AD. Its underlying mechanism was explored using an AD mouse model and splenocytes isolated from this model. Ta-EE ameliorated the AD symptoms without any toxicity and protected the skin of 2,4-dinitrochlorobenzene- (DNCB-) induced AD mice from damage and epidermal thickness. Ta-EE reduced the secreted levels of allergic and proinflammatory cytokines, including histamine, immunoglobulin E (IgE), interleukin- (IL-) 4, and interferon-gamma (IFN-*γ*) in the DNCB-induced AD mice. Ta-EE suppressed the mRNA expression of T helper 2-specific cytokines, IL-4 and IL-5, and the proinflammatory cytokine IFN-*γ* in the atopic dermatitis skin lesions of AD mice. Moreover, Ta-EE suppressed the mRNA expression of IL-4, IL-5, IFN-*γ*, and another proinflammatory cytokine, IL-12, in the Con A-stimulated splenocytes. It also suppressed IL-12 and IFN-*γ* in the LPS-stimulated splenocytes. Taken together, these results suggest that Ta-EE protects against the development of AD through the inhibition of mRNA expression of T helper 2-specific cytokines and other proinflammatory cytokines.

## 1. Introduction

Atopic dermatitis (AD) is the most common chronic allergic and inflammatory disease of the skin and often further precedes allergic diseases [1, 2]. Atopic dermatitis is characterized by epidermal barrier dysfunction, cutaneous inflammation, and pruritus (itch and rashes), which is a hallmark of AD. These symptoms lead to excoriations, inflammation, and disease worsening [3–5]. AD has been proven to be strongly associated with increased prevalence of comorbidities, including immunoglobulin E- (IgE-) related diseases (atopy), skin inflammation and infections, and mental disorders [6, 7]. The prevalence of AD is high in many countries, based on several studies with data from over one million children from 97 countries [8, 9]. The global prevalence of AD is approximately 15–30% in children and approximately 10% in adults [10, 11]. The pathogenesis of AD is complicated, involving the activation of inflammatory immune cells including T helper 2 cells, eosinophils, and macrophages. This activation results in the secretion of various proinflammatory cytokines. Although topical therapeutics is currently used for AD treatment, understanding the pathogenesis of AD is crucial for the development of more effective therapeutics and diagnostics. Therefore, efforts have been made to understand the pathogenesis of AD in order to develop better medications with new paradigms to prevent and treat AD.

Inflammation is a complex immune response that is mediated by inflammatory immune cells to protect the body from invading pathogens, including bacteria, viruses, fungi, and protozoa. It is characterized by several hallmark signs, including redness, swelling, heat, pain, and organ dysfunction [12–14]. Inflammation is a host defense mechanism; however, chronic inflammation is one of the major causes for a variety of human inflammatory and autoimmune diseases [15, 16]. Inflammatory responses are generally induced by innate immune cells, such as monocytes and macrophages, through activating intracellular inflammatory signaling cascades [16–20]. However, a number of studies have reported that inflammatory responses during AD pathogenesis are initiated through the activation of T helper 2 responses due to a breakdown in the balance between T helper 1 and 2 responses [1, 21]. The activation of T helper 2 responses induces the secretion of T helper 2-specific proinflammatory cytokines, including interleukin- (IL-) 4 and IL-5 [22, 23]. These cytokines subsequently induce the secretion of other types of proinflammatory cytokines, including IL-12 and IFN-*γ*, through activating eosinophils and T helper 1 cells, respectively [24, 25]. In addition, these inflammatory cytokines induce the release of allergic and inflammatory substances, such as immunoglobulin E (IgE) and histamine, by activating B-cells and mast cells, respectively [26].

*Tabebuia avellanedae* Lorentz ex Griseb (Bignoniaceae) is a tree belonging to the *Tabebuia* genus of the Bignoniaceae family, which is generally found in the tropical rain forests of certain South American countries including Brazil, Paraguay, and Northern Argentina. The inner bark of this tree, known as Taheebo or pau d'arco, has traditionally been used against various pathogenic and disease conditions [27–29]. A lot of effort has been made to identify and validate the active pharmacological compounds in this plant. Therefore, a number of compounds, including flavonoids, iridoids, coumarins, anthraquinone-2-carboxlic acid, cyclopentene derivatives, benzaldehyde derivatives, benzoic acid derivatives, *β*-lapachone, and quinones, including naphthoquinones, furanonaphthoquinones, and anthraquinones, have been extracted from *T. avellanedae* [30–36]. Some of these compounds have shown anti-infectious, anticancer, or anti-inflammatory activities [30, 34, 37–40]. Despite these studies, however, the pharmacological effect of *T. avellanedae* on inflammatory responses and inflammatory diseases and its underlying mechanisms are poorly understood.

In this study, we examined the pharmacological effects of the ethanol extract of *T. avellanedae* (Ta-EE, Tabetri) on the pathogenesis of AD. Furthermore, we studied the Ta-EE mechanism using a 2,4-dinitrochlorobenzene- (DNCB-) induced AD mouse model and splenocytes isolated from the DNCB-induced AD mice.

## 2. Materials and Methods

### 2.1. Materials

Ta-EE was kindly provided by Nutribiotech Co. Ltd. (Seoul, Korea). DNCB, concanavalin A (Con A), prednisolone, sodium dodecyl sulfate (SDS), hematoxylin, and eosin were purchased from Sigma Chemical Co. (St. Louis, MO, USA). Roswell Park Memorial Institute (RPMI) 1640, fetal bovine serum (FBS), phosphate-buffered saline (PBS), streptomycin, penicillin, and L-glutamine were purchased from Gibco (Grand Island, NY, USA). Enzyme-linked immunosorbent assay (ELISA) kits for histamine, immunoglobulin E (IgE), interleukin- (IL-) 4, and interferon-gamma (IFN-*γ*) were purchased from R&D Systems (Minneapolis, MN, USA). The TRI reagent® was purchased from the Molecular Research Center Inc. (Cincinnati, OH, USA). MuLV reverse transcriptase was purchased from Thermo Fisher Scientific (Waltham, MA, USA). Primers used for quantitative real-time polymerase chain reaction (PCR) were synthesized at Bioneer Inc. (Daejeon, Korea). The pPCRBIO SyGreen Mix for quantitative real-time PCR was purchased from PCR Biosystems Ltd. (London, United Kingdom).

### 2.2. Animals and Husbandry

NC/Nga mice (male, 5 weeks old) used for DNCB-induced atopic dermatitis were obtained from Daehan Biolink (Osong, Korea). The mice were housed in plastic cages (with five animals per cage) at room temperature with 12/12 h constant light/dark cycles. The mice were fed water and a custom pelleted diet *ad libitum* (Samyang, Daejeon, Korea). *In vivo* studies using these mice were conducted in accordance with the guideline of the Institutional Animal Care and Use Committee at Sungkyunkwan University.

### 2.3. Induction and Monitoring of DNCB-Induced AD in Mice

An AD mouse model was generated by treatment of the NC/Nga mice with DNCB, as previously described with a slight modification [41]. Briefly, 0.2 ml of 1% DNCB in acetone/olive oil (3 : 1) was applied for DNCB sensitization. After three days, 0.2 ml of 0.4% DNCB in acetone/olive oil (3 : 1) was repeatedly applied on the shaved skin of the dorsal area two times per week for four weeks. The severity of the atopic dermatitis skin lesions in the AD mice was monitored every week.

### 2.4. Measurement of Atopic Dermatitis Scores

The clinical severity scores of atopic dermatitis were defined on a scale of 0–8 as follows: 0 (none), 2 (mild), 4 (moderate), and 8 (severe) for five manifestations: itching, erythema/hemorrhage, and scaling/dryness. The scores were measured once per week from week 0 to 4 by three trained persons blinded to the experiment.

### 2.5. Measurement of Body Weight

Body weight of the mice in all experimental groups was measured (by trained persons who were blinded to the experiment) once a week from week 0 to week 4 using a custom scale (Mettler Toledo, Columbus, OH, USA).

### 2.6. Hematoxylin and Eosin (H&E) Staining

After euthanasia at the end of the study (week 4), the atopic dermatitis skin lesions were excised and fixed in 10% formalin solution before paraffin processing. Formalin-fixed sections of skin tissues (4 *μ*m thickness) in each experimental group were stained with H&E to observe the histopathological changes [42].

### 2.7. Measurement of Epidermal Thickness

In order to quantify changes in the epidermal thickness, the skin tissues (the atopic dermatitis skin lesions and normal skins) stained with H&E were photographed. The epidermal thickness was determined by counting the pixels of the epidermis areas in the photos using ImageJ software (National Institutes of Health, Bethesda, MD).

### 2.8. Enzyme-Linked Immunosorbent Assay (ELISA)

The secreted levels of histamine, IgE, IL-4, and IFN-*γ* in the sera of the DNCB-induced AD mice were orally administered with either Ta-EE (0, 60, 120, and 240 mg/kg) or prednisolone (3 mg/kg). The levels were determined by ELISA at week 0, 2, and 4 according to the manufacturer's instructions.

### 2.9. Quantitative Real-Time Polymerase Chain Reaction (PCR)

In order to determine the mRNA expression levels of IL-4, IL-5, IFN-*γ*, and IL-12, the total RNA was extracted from the atopic dermatitis skin lesions of the DNCB-induced AD mice orally administered with either Ta-EE (0, 60, 120, and 240 *μ*g/ml) or prednisolone (3 mg/kg). The total splenocytes prepared from the normal mice were treated with Ta-EE (0, 75, 150, and 300 *μ*g/ml) for 30 min, followed by treatment with either Con A (10 *μ*g/ml) or LPS (1 *μ*g/ml) for 6 h using TRI reagent according to the manufacturer's instruction. The total cDNA was immediately synthesized from 1 *μ*g of total RNA using MuLV reverse transcriptase according to the manufacturer's instruction. Quantitative real-time PCR was conducted as previously described [16, 43]. The nucleic acid sequences of the primers used for quantitative real-time PCR are listed in Table 1.

### 2.10. Preparation of Cell Lysate and Immunoblotting Analysis

Skin tissues were used to obtain tissue lysates as reported previously. Tissue lysates were immunoblotted, and total protein and phosphoprotein levels of Syk, IKK, I*κ*B*α*, p65, and *β*-actin were determined, as previously reported [44, 45].

### 2.11. Statistical Analysis

The data acquired from this study are presented as means and standard deviation of at least three independent experiments. All results were analyzed using the ANOVA/Scheffe's post hoc test and Kruskal-Wallis/Mann–Whitney *U* tests. *P* values < 0.05 were considered statistically significant. Statistical analyses were performed using the SPSS program.

## 3. Results and Discussion

Several studies have suggested that compounds isolated from *T. avellanedae* and Taheebo extract have anti-inflammatory activities [34–36, 38, 40, 46, 47]. However, the pharmacological mechanisms of *T. avellanedae* on inflammation and inflammatory diseases remain poorly understood. There has been no study regarding the effect of *T. avellanedae* on the pathogenesis of AD, which is one of the most common inflammatory and allergic skin diseases. Therefore, the present study investigates the pharmacological effect of Ta-EE on the pathogenesis of AD using a DNCB-induced AD mouse model and examines the cellular mechanism of Ta-EE-mediated anti-inflammatory activity using splenocytes isolated from the DNCB-induced AD mice.

**Figure 1:**
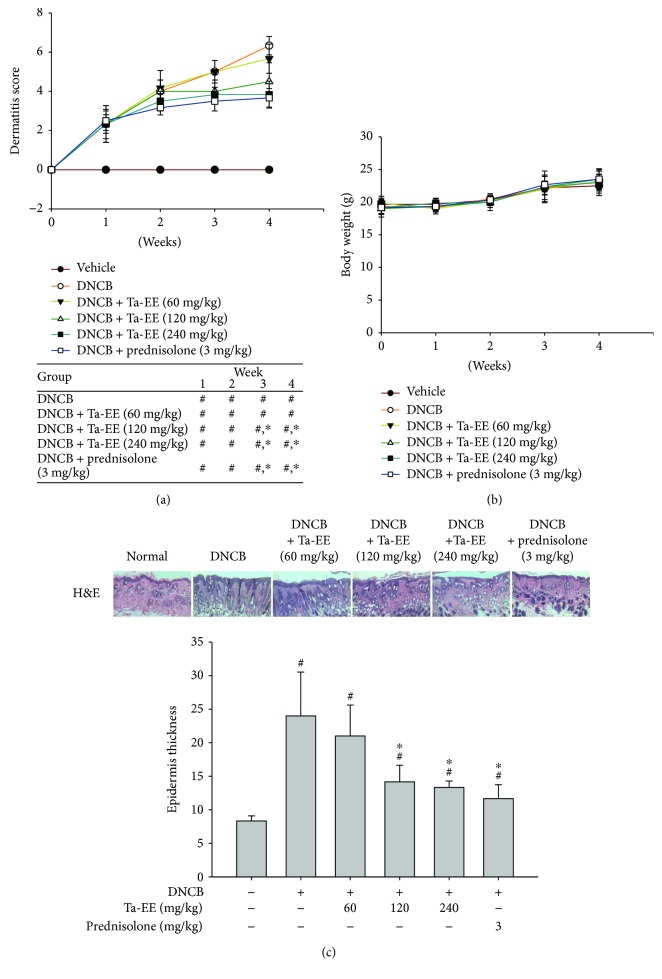
Ta-EE protected against AD symptoms in DNCB-induced AD mice. DNCB-induced AD mice were orally administered with either Ta-EE (0, 60, 120, and 240 mg/kg) or prednisolone (3 mg/kg) once a day for 4 weeks. (a) Dermatitis scores and (b) body weight of the AD mice were determined once a week from week 0 to week 4. ((c), upper panel) DNCB-induced AD mice were orally administered with either Ta-EE (0, 60, 120, and 240 mg/kg) or prednisolone (3 mg/kg) once a day for 4 weeks. The atopic dermatitis skin was excised from the AD mice after the animals were sacrificed at week 4. The atopic dermatitis skin lesions were stained with H&E. ((c), lower panel) The epidermal thickness of these AD mice was measured and plotted using ImageJ software. ^∗^*P* < 0.05 versus a control group; ^#^*P* < 0.05 versus a normal group.

We first examined whether Ta-EE has *in vivo* pharmacological effects on the pathogenesis of AD using DNCB-induced AD mice. Ta-EE ameliorated the AD-like symptoms and significantly decreased the dermatitis scores in the DNCB-induced AD mice in a dose-dependent manner (Figure 1(a)). The anti-AD effect of Ta-EE at doses of 120 and 240 mg/kg was statistically comparable to that of prednisolone (which is currently used as an anti-inflammatory drug) (Figure 1(a)). This finding suggests that Ta-EE may be a promising candidate to treat AD with a comparable pharmacological effect to a currently approved anti-inflammatory drug. Although Ta-EE significantly decreased the dermatitis scores in the DNCB-induced AD mice, it was well tolerated at all doses and did not cause any weight loss (Figure 1(b)) or abnormal behaviors (data not shown) during the entire experimental period. These findings indicate that Ta-EE has no or undetectable *in vivo* toxicity or adverse effects at the doses that we tested in this study. The pharmacological effect of Ta-EE on AD pathogenesis was further evaluated by histopathological staining of the skin epidermal tissues from the DNCB-induced AD mice. The H&E staining showed that Ta-EE markedly protected the skin from damage in the DNCB-induced AD mice (Figure 1(c); upper panel). Lichenification is one of the symptomatic hallmarks of AD that is characterized by skin thickening of the epidermal tissues [48]. Therefore, the effect of Ta-EE on epidermal thickness was examined. Ta-EE significantly reduced the epidermal thickness induced by DNCB in AD mice in a dose-dependent manner (Figure 1(c); lower panel). In accordance with the result of the dermatitis scores, the suppressive effect of Ta-EE on epidermal thickness was statistically comparable to that of prednisolone at doses of 120 and 240 mg/kg (Figure 1(c); lower panel). These results strongly indicate that Ta-EE has *in vivo* pharmacological effects to ameliorate AD symptoms by reducing dermatitis scores (which represent various AD symptoms, as well as epidermal thickness in the DNCB-induced AD mice).

**Figure 2:**
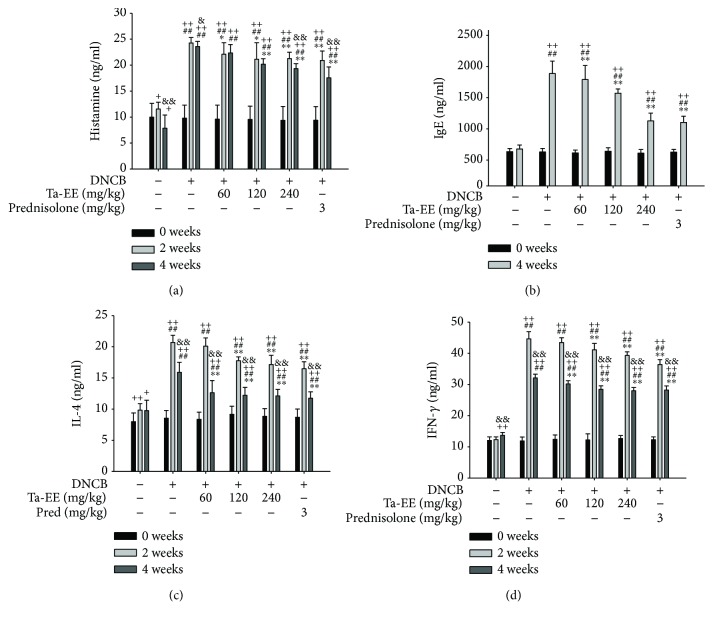
Effects of Ta-EE on the serum levels of allergic and proinflammatory substances and cytokines in DNCB-induced AD mice. DNCB-induced AD mice were orally administered with either Ta-EE (0, 60, 120, and 240 mg/kg) or prednisolone (3 mg/kg) once a day for 4 weeks. The secreted levels of (a) histamine, (c) IL-4, and (d) IFN-*γ* in the sera of AD mice were measured at weeks 0, 2, and 4. The secreted level of (b) IgE in the sera of AD mice was measured at weeks 0 and 4. ^∗^*P* < 0.05 and ^∗∗^*P* < 0.005 versus a control group; ^##^*P* < 0.005 versus a normal group; ^+^*P* < 0.05 and ^++^*P* < 0.005 week 0 versus week 2 or 4; ^&^*P* < 0.05 and ^&&^*P* < 0.005 week 2 versus week 4.

The pathogenesis of AD is complex and multifactorial. The onset and progression of AD were originally reported to be attributed to the imbalance between T helper 1 and T helper 2 responses [1]. An increase in the numbers of T helper 2 cells and the induction of T helper 2 cell-mediated immune responses [21] leads to the production of T helper 2-specific cytokines such as IL-4 and IL-5 [22, 23]. These T helper 2-specific cytokines not only stimulate eosinophils to secrete IL-12 (an activator of T helper 1 cells) to release IFN-*γ* but also induce the secretion of IgE in B-cells. IFN-*γ* is responsible for the exacerbation of skin inflammation [24, 25], while IgE secretion activates mast cells to release allergic and inflammatory mediators such as histamine [26]. Several studies have suggested that the serum level of IgE is high in AD patients and that AD pathogenesis may be strongly correlated with elevated serum levels of IgE, which is similar to that of allergic diseases such as asthma and allergic rhinitis [49–52]. Therefore, given the evidence from previous studies regarding AD pathogenesis, we further investigated the *in vivo* pharmacological effects of Ta-EE on AD pathogenesis by examining the secreted levels of allergic and proinflammatory substances and cytokines including histamine, IgE, IFN-*γ*, and IL-4 in the sera of DNCB-induced AD mice. The Ta-EE decreased the serum level of histamine at both weeks 2 and 4 (Figure 2(a)). Ta-EE also reduced the serum level of IgE in a dose-dependent manner, while there was no induction of IgE level during Ta-EE treatment in normal mice (data not shown). The inhibitory effect of Ta-EE was comparable to that of prednisolone at a dose of 240 mg/kg (Figure 2(b)). Moreover, Ta-EE significantly decreased the serum levels of IL-4 (Figure 2(c)) and IFN-*γ* (Figure 2(d)) in a dose-dependent manner. The inhibitory effect of Ta-EE on IL-4 production was comparable to that of prednisolone at a dose of 240 mg/kg (Figure 2(c)). These results suggest that Ta-EE exerts pharmacological effects on the pathogenesis of AD by reducing the secreted levels of allergic and proinflammatory substances and cytokines in the sera of DNCB-induced AD mice.

**Figure 3:**
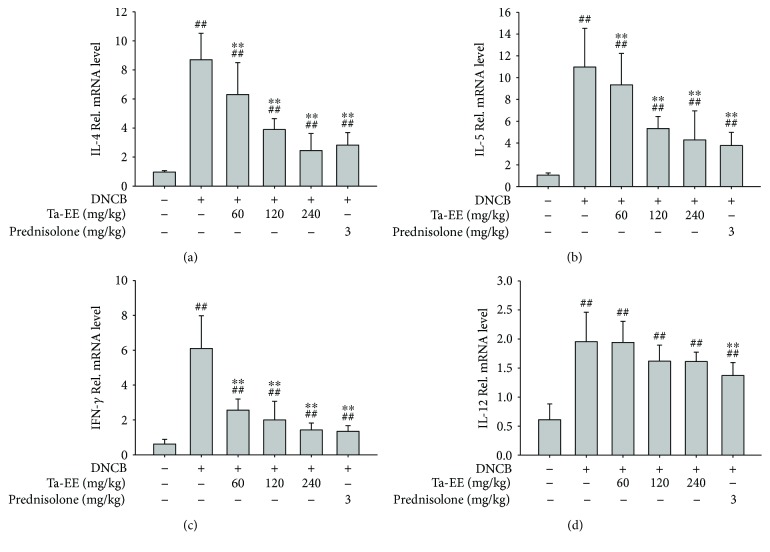
Effects of Ta-EE on mRNA expression of proinflammatory cytokines in the atopic dermatitis skin lesions of DNCB-induced AD mice. DNCB-induced AD mice were orally administered with either Ta-EE (0, 60, 120, and 240 mg/kg) or prednisolone (3 mg/kg) once a day for 4 weeks. The atopic dermatitis skins were excised from the AD mice after sacrifice at week 4. The mRNA expression levels of (a) IL-4, (b) IL-5, (c) IFN-*γ*, and (d) IL-12 in the atopic dermatitis skins were determined using quantitative real-time PCR. ^∗∗^*P* < 0.005 versus a control group; ^##^*P* < 0.005 versus a normal group.

We further investigated the effects of Ta-EE on the expression of proinflammatory and T helper 2-specific cytokines in a transcriptional level by quantitative real-time PCR in the DNCB-induced AD mice. First, atopic dermatitis skin lesions were excised from the mice in each group. The effect of Ta-EE on the gene expression of proinflammatory and T helper 2-specific cytokines was examined in the total cells prepared from the atopic dermatitis skin lesions. Ta-EE significantly downregulated mRNA expression of IL-4 (Figure 3(a)), IL-5 (Figure 3(b)), and IFN-*γ* (Figure 3(c)) in dose-dependent manner for all the three. The suppressive effect of Ta-EE was comparable to that of prednisolone at 240 mg/kg (Figures 3(a)–3(c)). However, Ta-EE did not downregulated mRNA expression of IL-12, while prednisolone showed the suppressive effect on IL-12 mRNA expression (Figure 3(d)). The reason why Ta-EE, unlike the other cytokines, did not downregulate mRNA expression of IL-12 is unclear. These results suggest that Ta-EE effectively suppresses the expression of T helper 2-specific cytokines in a transcriptional level induced at the skin inflammatory lesions during AD pathogenesis. Its suppressive effect is comparable to that of the currently available anti-inflammatory drug prednisolone.

**Figure 4:**
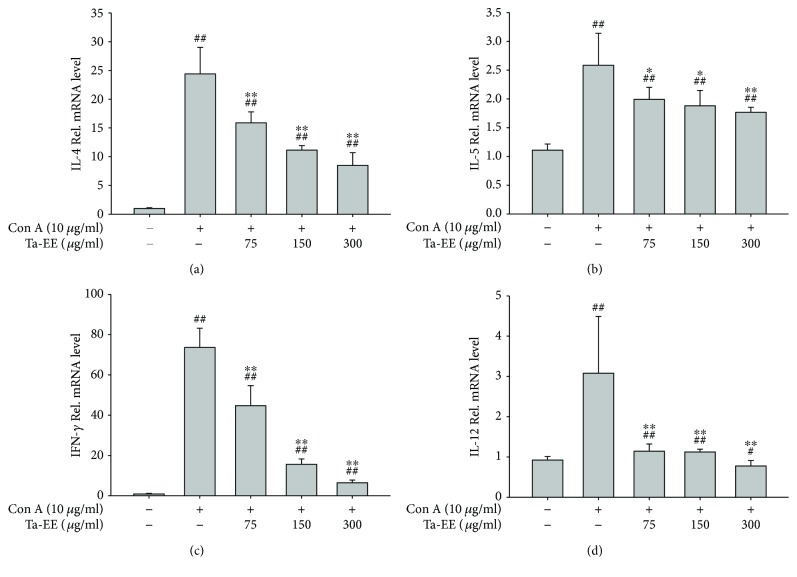
Effects of Ta-EE on mRNA expression of proinflammatory cytokines in Con A-stimulated splenocytes. The total splenocytes isolated from the spleens of normal mice pretreated with Ta-EE (0, 75, 150, and 300 *μ*g/ml) for 30 min were treated with Con A (10 *μ*g/ml) for 6 h. The mRNA expression levels of (a) IL-4, (b) IL-5, (c) IFN-*γ*, and (d) IL-12 in the splenocytes were determined using quantitative real-time PCR. ^∗^*P* < 0.05 and ^∗∗^*P* < 0.005 versus a control group; ^#^*P* < 0.05 and ^##^*P* < 0.005 versus a normal group.

The spleen is the secondary lymphoid organ where effector T-cells activate humoral and cellular immune responses. Therefore, the effect of Ta-EE on mRNA expression of proinflammatory and T helper 2-specific cytokines was also examined in the splenocytes. The total splenocytes prepared from mice were stimulated with Con A, which is a known T-cell mitogen [53]. The effect of Ta-EE on mRNA expression of IL-4, IL-5, IFN-*γ*, and IL-12 in the splenocytes was examined by quantitative real-time PCR. As expected, Ta-EE markedly downregulated the mRNA expression of IL-4 (Figure 4(a)), IL-5 (Figure 4(b)), IFN-*γ* (Figure 4(c)), and IL-12 (Figure 4(d)) in a dose-dependent manner in the Con A-stimulated splenocytes.

**Figure 5:**
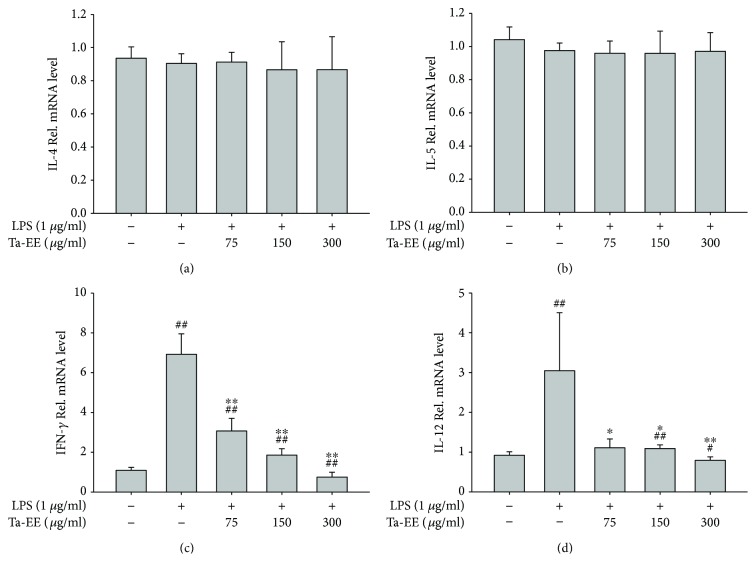
Effects of Ta-EE on mRNA expression of proinflammatory cytokines in LPS-stimulated splenocytes. The total splenocytes isolated from the spleens of normal mice were pretreated with Ta-EE (0, 75, 150, and 300 *μ*g/ml) for 30 min with LPS (1 *μ*g/ml) for 6 h. The mRNA expression levels of (a) IL-4, (b) IL-5, (c) IFN-*γ*, and (d) IL-12 in the splenocytes were determined by quantitative real-time PCR. ^∗^*P* < 0.05 and ^∗∗^*P* < 0.005 versus a control group; ^#^*P* < 0.05 and ^##^*P* < 0.005 versus a normal group.

The effects of Ta-EE on mRNA expression of proinflammatory and T helper 2-specific cytokines were further examined in the splenocytes stimulated with lipopolysaccharide (LPS). LPS is a specific ligand of toll-like receptor 4 (TLR4). Ta-EE did not suppress the mRNA expression of IL-4 (Figure 5(a)) or IL-5 (Figure 5(b)). These cytokines were also not induced by LPS in the splenocytes. This result is expected because IL-4 and IL-5 are the cytokines secreted from the T helper 2 cells, which do not express TLR4 on their surfaces. In contrast, Ta-EE significantly suppressed mRNA expression of IFN-*γ* (Figure 5(c)) and IL-12 (Figure 5(d)) in the LPS-stimulated splenocytes in a dose-dependent manner. IL-12 is known to be secreted from eosinophils activated by T helper 2-specific cytokines [24, 25]. However, the expression of these T helper 2-specific cytokines was not induced in the LPS-stimulated splenocytes (Figures 5(a) and 5(b)), suggesting that IL-12 could be expressed and secreted in the splenocytes in a T helper 2-specific cytokine-independent manner. Interestingly, it has been reported that eosinophils in the spleen express TLR4 [54, 55]. This expression pattern suggests that Ta-EE suppressed mRNA expression of IL-12 induced by LPS in the eosinophils recruited in the spleen during AD pathogenesis. This suppressive activity of Ta-EE is T helper 2 specific and cytokine independent. Ta-EE also suppressed mRNA expression of IFN-*γ* in the LPS-stimulated splenocytes (Figure 5(c)). As previously discussed, IL-12 induces the expression and secretion of IFN-*γ* in T helper 1 cells [24, 25]. Therefore, this result suggests that Ta-EE suppressed mRNA expression of IFN-*γ* induced by LPS in the T helper 1 cells in response to IL-12, a T helper 1 cell activator secreted from the activated eosinophils in the spleen during AD pathogenesis.

**Figure 6:**
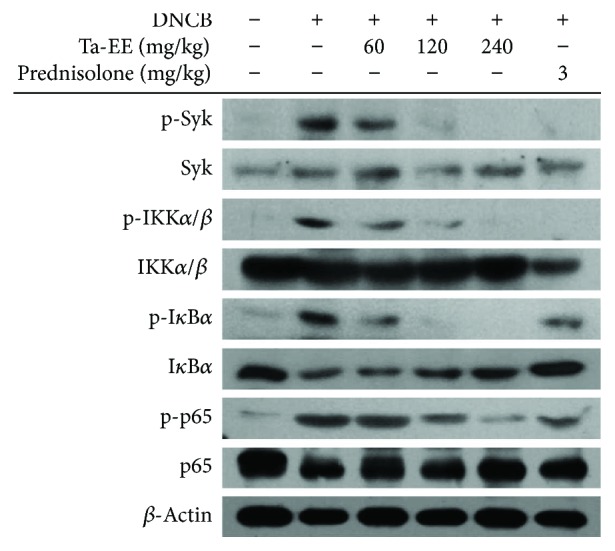
Effect of Ta-EE on the activation of inflammatory signaling molecules. Phospho- or total form levels of p65/NF-*κ*B, I*κ*B*α*, IKK*α*/*β*, Syk, and *β*-actin from skin tissue of DNCB-induced AD mice were detected by immunoblotting analysis with phospho-specific or total protein antibodies from total cell lysates.

Finally, to understand which signaling enzymes contributed to the suppression of Ta-EE on various AD symptoms, the levels of phosphoproteins and total proteins such as Syk, IKK, IκB*α*, and p65, one of the NF-*κ*B subunits, known as critical factors in AD pathogenesis [56, 57] and reported to be targeted by anthraquinone compounds enriched in this plant [36, 58], were determined from the skin area in AD mouse. As we expected, it was found that the phosphoproteins of these proteins were strongly suppressed in a dose-dependent manner without altering total protein levels (Figure 6). Therefore, these results might suggest that the suppression of the Syk/NF-*κΒ* pathway could be linked to blockade of inflammatory symptoms in AD. Since the Syk and NF-*κ*B pathways are major inflammation-inducing signaling in macrophages, it is assumed a possibility that activated macrophages or dendritic cells could play critical roles in the earlier inflammatory process in AD models. In the following experiments, this possibility will be further evaluated to understand target cells in Ta-EE-mediated antiatopic dermatitis activity.

## 4. Conclusions

**Figure 7:**
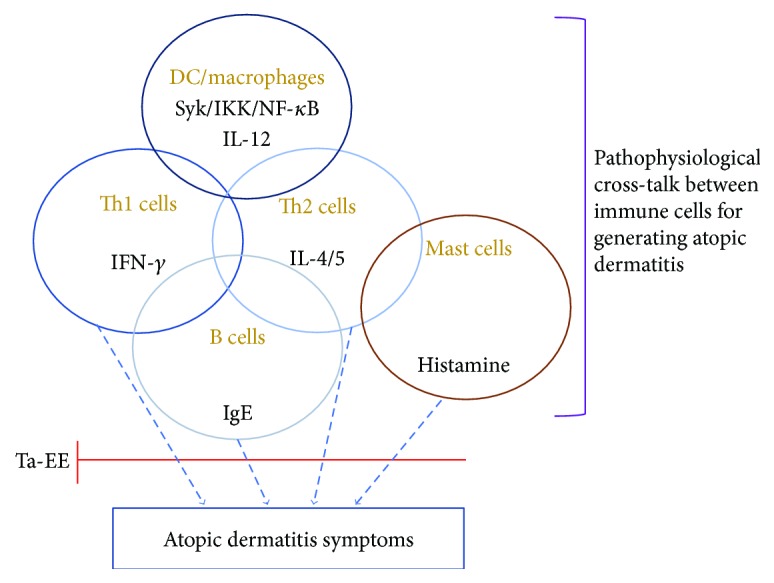
Putative inhibitory pathway of Ta-EE-mediated antidermatitis activity.

This study explored the *in vivo* pharmacological effects of Ta-EE on the pathogenesis of AD using a DNCB-induced AD-like mouse model and isolated splenocytes. Ta-EE effectively delayed the onset and progression of AD and ameliorated AD symptoms without any toxicities and adverse effects in the DNCB-induced AD mice. The cellular and molecular mechanism studies showed that the pharmacological effects of Ta-EE were attributable to the suppression of allergic and inflammatory substances and cytokines during the pathogenesis of AD as summarized in Figure 7. Ta-EE inhibited the production of allergic substances (such as histamine and IgE), proinflammatory cytokines (including IFN-*γ*), and the T helper 2-specific cytokine (IL-4) in the DNCB-induced AD mice. In addition, Ta-EE suppressed the expression of T helper 2-specific cytokines, such as IL-4 and IL-5 and proinflammatory cytokines, such as IFN-*γ* and IL-12 in a transcriptional level in the atopic dermatitis skin lesions of the AD mice and total splenocytes. Taken together, this study reports the pharmacological effects of *T. avellanedae* extract on the pathogenesis of AD for the first time and provides evidence of a correlation between inflammatory/allergic responses and AD pathogenesis. Therefore, these findings could improve our understanding of AD pathogenesis at the cellular and molecular levels and provide insight into the development of novel promising medications or dietary supplements/health functional foods using medicinal plants to prevent and treat various inflammatory and allergic diseases. Indeed, we are currently undertaking a clinical trial with this extract to prove its efficacy in the human body.

## Figures and Tables

**Table 1:** Nucleic acid sequences of the primers used for quantitative real-time PCR.

Target		Sequence (5′ to 3′)
IL-4	Forward	GGTCTCAACCCCCAGCTAGT
	Reverse	GCCCATGATCTCTCTCAAGT

IL-5	Forward	TCTTCAGTATGTCTAGCCCCTG
	Reverse	CTCTGTTGACAAGCAATGAGACG

IFN-*γ*	Forward	GGGTTGTTGACCTCAAACTT
	Reverse	CAGGCCATCAGCAACAACAT

IL-12	Forward	TGAACTGGCGTTGGAAGC
	Reverse	GCGGGTCTGGTTTGATGA

GAPDH	Forward	CAATGAATACGGCTACAGCAAC
	Reverse	AGGGAGATGCTCAGTGTTGG

## References

[1] Leung D. Y., Boguniewicz M., Howell M. D., Nomura I., Hamid Q. A. (2004). New insights into atopic dermatitis. *The Journal of Clinical Investigation*.

[2] Boguniewicz M., Leung D. Y. (2010). Recent insights into atopic dermatitis and implications for management of infectious complications. *The Journal of Allergy and Clinical Immunology*.

[3] Silverberg J. I., Nelson D. B., Yosipovitch G. (2016). Addressing treatment challenges in atopic dermatitis with novel topical therapies. *Journal of Dermatological Treatment*.

[4] Hong J., Buddenkotte J., Berger T. G., Steinhoff M. (2011). Management of itch in atopic dermatitis. *Seminars in Cutaneous Medicine and Surgery*.

[5] Yosipovitch G., Papoiu A. D. (2008). What causes itch in atopic dermatitis?. *Current Allergy and Asthma Reports*.

[6] Simpson E. L., Eichenfield L. F., Ellis C. N., Mancini A. J., Paller A. S. (2012). Current issues in atopic comorbidities and preventing the atopic march. *Seminars in Cutaneous Medicine and Surgery*.

[7] Silverberg J. I., Simpson E. L. (2013). Association between severe eczema in children and multiple comorbid conditions and increased healthcare utilization. *Pediatric Allergy and Immunology*.

[8] Williams H., Stewart A., von Mutius E., Cookson W., Ross Anderson H. (2008). Is eczema really on the increase worldwide?. *The Journal of Allergy and Clinical Immunology*.

[9] Odhiambo J. A., Williams H. C., Clayton T. O., Robertson C. F., Asher M. I., ISAAC Phase Three Study Group (2009). Global variations in prevalence of eczema symptoms in children from ISAAC phase three. *The Journal of Allergy and Clinical Immunology*.

[10] Simpson E. L. M. M., Irvine A. D. M., Eichenfield L. F. M., Friedlander S. F. M. (2016). Update on epidemiology, diagnosis, and disease course of atopic dermatitis. *Seminars in Cutaneous Medicine and Surgery*.

[11] Udkoff J., Waldman A., Ahluwalia J., Borok J., Eichenfield L. F. (2017). Current and emerging topical therapies for atopic dermatitis. *Clinics in Dermatology*.

[12] Janeway C. A., Medzhitov R. (2002). Innate immune recognition. *Annual Review of Immunology*.

[13] Yi Y. S. (2016). Folate receptor-targeted diagnostics and therapeutics for inflammatory diseases. *Immune Network*.

[14] Kim J. H., Yoo B. C., Yang W. S., Kim E., Hong S., Cho J. Y. (2016). The role of protein arginine methyltransferases in inflammatory responses. *Mediators in Inflammation*.

[15] Kaur M., Singh M., Silakari O. (2013). Inhibitors of switch kinase ‘spleen tyrosine kinase’ in inflammation and immune-mediated disorders: a review. *European Journal of Medicinal Chemistry*.

[16] Baek K. S., Yi Y. S., Son Y. J. (2016). *In vitro* and *in vivo* anti-inflammatory activities of Korean red ginseng-derived components. *Journal of Ginseng Research*.

[17] Yi Y. S., Son Y. J., Ryou C., Sung G. H., Kim J. H., Cho J. Y. (2014). Functional roles of Syk in macrophage-mediated inflammatory responses. *Mediators in Inflammation*.

[18] Yang Y., Kim S. C., Yu T. (2014). Functional roles of p38 mitogen-activated protein kinase in macrophage-mediated inflammatory responses. *Mediators of Inflammation*.

[19] Yu T., Yi Y. S., Yang Y., Oh J., Jeong D., Cho J. Y. (2012). The pivotal role of TBK1 in inflammatory responses mediated by macrophages. *Mediators of Inflammation*.

[20] Byeon S. E., Yi Y. S., Oh J., Yoo B. C., Hong S., Cho J. Y. (2012). The role of Src kinase in macrophage-mediated inflammatory responses. *Mediators of Inflammation*.

[21] Akdis M., Trautmann A., Klunker S. (2003). T helper (Th) 2 predominance in atopic diseases is due to preferential apoptosis of circulating memory/effector Th1 cells. *The FASEB Journal*.

[22] Forbes E., van Panhuys N., Min B., Le Gros G. (2010). Differential requirements for IL-4/STAT6 signalling in CD4 T-cell fate determination and Th2-immune effector responses. *Immunology & Cell Biology*.

[23] Maeda S., Yanagihara Y. (2001). Inflammatory cytokines (IL-4, IL-5 and IL-13). *Nihon Rinsho Japanese Journal of Clinical Medicine*.

[24] Wegmann M. (2009). Th2 cells as targets for therapeutic intervention in allergic bronchial asthma. *Expert Review of Molecular Diagnostics*.

[25] Grewe M., Czech W., Morita A. (1998). Human eosinophils produce biologically active IL-12: implications for control of T cell responses. *Journal of Immunology*.

[26] Ji G. E. (2009). Probiotics in primary prevention of atopic dermatitis. *Forum of Nutrition*.

[27] Casinovi C. G., Marini Bettolo G. B., Limaog D. A., Daliamaia M. E., d’ Albuquerque I. L. (1963). Quinones isolated from the wood of Tabebuia avellanedae Lor. Ex Griseb. *Rendiconti-Istituto Superiore di Sanità*.

[28] de Santana C. F., de Lima O., d’ Albuquerque I. L., Lacerda A. L., Martins D. G. (1968). Antitumoral and toxicological properties of extracts of bark and various wood components of Pau d’arco (*Tabebuia avellanedae*). *Revista do Instituto de Antibióticos, Universidade Federal de Pernambuco*.

[29] Woo H. J., Choi Y. H. (2005). Growth inhibition of A549 human lung carcinoma cells by *β*-lapachone through induction of apoptosis and inhibition of telomerase activity. *International Journal of Oncology*.

[30] Choi B. T., Cheong J., Choi Y. H. (2003). Beta-Lapachone-induced apoptosis is associated with activation of caspase-3 and inactivation of NF-kappaB in human colon cancer HCT-116 cells. *Anti-Cancer Drugs*.

[31] Pereira E. M., Machado Tde B., Leal I. C. (2006). Tabebuia avellanedae naphthoquinones: Activity against methicillin-resistant staphylococcal strains, cytotoxic activity and in vivo dermal irritability analysis. *Annals of Clinical Microbiology and Antimicrobials*.

[32] Kim S. O., Kwon J. I., Jeong Y. K., Kim G. Y., Kim N. D., Choi Y. H. (2007). Induction of Egr-1 is associated with anti-metastatic and anti-invasive ability of *β*-lapachone in human hepatocarcinoma cells. *Bioscience, Biotechnology, and Biochemistry*.

[33] Kung H. N., Chien C. L., Chau G. Y., Don M. J., Lu K. S., Chau Y. P. (2007). Involvement of NO/cGMP signaling in the apoptotic and anti-angiogenic effects of *β*-lapachone on endothelial cells in vitro. *Journal of Cellular Physiology*.

[34] Xu J., Wagoner G., Douglas J. C., Drew P. D. (2013). *β*-Lapachone ameliorization of experimental autoimmune encephalomyelitis. *Journal of Neuroimmunology*.

[35] Zhang L., Hasegawa I., Ohta T. (2016). Anti-inflammatory cyclopentene derivatives from the inner bark of *Tabebuia avellanedae*. *Fitoterapia*.

[36] Park J. G., Son Y. J., Kim M. Y., Cho J. Y. (2016). Syk and IRAK1 contribute to immunopharmacological activities of anthraquinone-2-carboxlic acid. *Molecules*.

[37] Machado T. B., Pinto A. V., Pinto M. C. (2003). In vitro activity of Brazilian medicinal plants, naturally occurring naphthoquinones and their analogues, against methicillin-resistant *Staphylococcus aureus*. *International Journal of Antimicrobial Agents*.

[38] Awale S., Kawakami T., Tezuka Y., Ueda J. Y., Tanaka K., Kadota S. (2005). Nitric oxide (NO) production inhibitory constituents of *Tabebuia avellanedae* from Brazil. *Chemical and Pharmaceutical Bulletin*.

[39] Bohler T., Nolting J., Gurragchaa P. (2008). *Tabebuia avellanedae* extracts inhibit IL-2-independent T-lymphocyte activation and proliferation. *Transplant Immunology*.

[40] Lee E. J., Ko H. M., Jeong Y. H., Park E. M., Kim H. S. (2015). *β*-Lapachone suppresses neuroinflammation by modulating the expression of cytokines and matrix metalloproteinases in activated microglia. *Journal of Neuroinflammation*.

[41] Choi J. H., Song Y. S., Lee H. J., Kim G. C., Hong J. W. (2017). The topical application of low-temperature argon plasma enhances the anti-inflammatory effect of Jaun-ointment on DNCB-induced NC/Nga mice. *BMC Complementary & Alternative Medicine*.

[42] Lu K. H., Weng C. Y., Chen W. C., Sheen L. Y. (2017). Ginseng essence, a medicinal and edible herbal formulation, ameliorates carbon tetrachloride-induced oxidative stress and liver injury in rats. *Journal of Ginseng Research*.

[43] Park J. G., Kang W. S., Park K. T., Aravinthan A., Kim J. H., Cho J. Y. (2016). Anticancer effect of joboksansam, Korean wild ginseng germinated from bird feces. *Journal of Ginseng Research*.

[44] Park J. G., Son Y. J., Aravinthan A., Kim J. H., Cho J. Y. (2016). Korean red ginseng water extract arrests growth of xenografted lymphoma cells. *Journal of Ginseng Research*.

[45] Hossen M. J., Hong Y. D., Baek K. S. (2017). *In vitro* antioxidative and anti-inflammatory effects of the compound K-rich fraction BIOGF1K, prepared from *Panax ginseng*. *Journal of Ginseng Research*.

[46] Byeon S. E., Chung J. Y., Lee Y. G., Kim B. H., Kim K. H., Cho J. Y. (2008). *In vitro* and *in vivo* anti-inflammatory effects of taheebo, a water extract from the inner bark of *Tabebuia avellanedae*. *Journal of Ethnopharmacology*.

[47] Lee M. H., Choi H. M., Hahm D. H. (2012). Analgesic and anti-inflammatory effects in animal models of an ethanolic extract of taheebo, the inner bark of Tabebuia avellanedae. *Molecular Medicine Reports*.

[48] Hyung K. E., Kim S. J., Jang Y. W. (2017). Therapeutic effects of orally administered CJLP55 for atopic dermatitis via the regulation of immune response. *The Korean Journal of Physiology & Pharmacology*.

[49] Kapoor R., Menon C., Hoffstad O., Bilker W., Leclerc P., Margolis D. J. (2008). The prevalence of atopic triad in children with physician-confirmed atopic dermatitis. *Journal of the American Academy of Dermatology*.

[50] Leung D. Y., Bieber T. (2003). Atopic dermatitis. *The Lancet*.

[51] Spergel J. M. (2010). From atopic dermatitis to asthma: the atopic march. *Annals of Allergy, Asthma & Immunology*.

[52] Kabashima K. (2013). New concept of the pathogenesis of atopic dermatitis: interplay among the barrier, allergy, and pruritus as a trinity. *Journal of Dermatological Science*.

[53] Dwyer J. M., Johnson C. (1981). The use of concanavalin A to study the immunoregulation of human T cells. *Clinical & Experimental Immunology*.

[54] Phipps S., Lam C. E., Mahalingam S. (2007). Eosinophils contribute to innate antiviral immunity and promote clearance of respiratory syncytial virus. *Blood*.

[55] Kvarnhammar A. M., Cardell L. O. (2012). Pattern-recognition receptors in human eosinophils. *Immunology*.

[56] Kim W. J., Cha H. S., Lee M. H., Kim S. Y., Kim S. H., Kim T. J. (2016). Effects of Cymbidium root ethanol extract on atopic dermatitis. *Evidence-based Complementary and Alternative Medicine*.

[57] Nam S. T., Park Y. H., Kim H. W. (2017). Suppression of IgE-mediated mast cell activation and mouse anaphylaxis via inhibition of Syk activation by 8-formyl-7-hydroxy-4-methylcoumarin, 4*μ*8C. *Toxicology and Applied Pharmacology*.

[58] Park J. G., Kim S. C., Kim Y. H. (2016). Anti-inflammatory and antinociceptive activities of anthraquinone-2-carboxylic acid. *Mediators of Inflammation*.

